# Impact of Lewy body and limbic-predominant TDP-43 neuropathology on cognitive and neuropsychiatric trajectory in Alzheimer’s disease: a retrospective neuropathological study

**DOI:** 10.3389/fnagi.2026.1724274

**Published:** 2026-04-17

**Authors:** Jarok Koo, Hyungjin Lee, Yunyeong Choi, Kyoungwon Baik, Mincheol Park, Alzheimer’s Disease Neuroimaging Initiative

**Affiliations:** 1 Chung-Ang University College of Medicine, Seoul, Republic of Korea; 2 Anam Hospital, Korea University College of Medicine, Seoul, Republic of Korea; 3 Department of Neurology, Chung-Ang University College of Medicine, Seoul, Republic of Korea; 4 Yonsei University College of Medicine, Seoul, Republic of Korea

**Keywords:** Alzheimer’s disease, Lewy body disease, limbic-predominant age-related TDP-43 encephalopathy, prognosis, TDP-43

## Abstract

**Introduction:**

Alzheimer’s disease (AD) is the leading cause of neurodegenerative dementia, and mixed neuropathological changes including Lewy body (LB-NC) and TDP-43 (LATE-NC) are commonly observed in patients with AD. We examined the baseline cross-sectional and longitudinal effects of these co-pathologies on cognitive and neuropsychiatric trajectories.

**Methods:**

We investigated 77 participants who had available autopsy data and showed intermediate to high levels of AD neuropathological change from the ADNI database. Participants were categorized based on the presence or absence of LB-NC or LATE-NC. The impact of LB-NC and LATE-NC on baseline and longitudinal clinical features was assessed using linear regression and linear mixed-effects models, respectively.

**Results:**

Thirty-eight (49.4%) had LB-NC, and 39 (50.6%) had LATE-NC. At baseline, the presence of LB-NC was not associated with cognitive function or neuropsychiatric symptoms, whereas the presence of LATE-NC was associated with better trail-making test performance and less severe sleep disturbance. Longitudinally, the presence of LB-NC was associated with faster cognitive decline in global cognitive function, memory, language, and executive function, whereas the presence of LATE-NC was associated with a slower decline in language function. Neither LB-NC nor LATE-NC influenced the longitudinal trajectory of neuropsychiatric symptoms.

**Conclusion:**

Among patients with pathologically confirmed AD, the presence of LB-NC accelerated cognitive decline, whereas the presence of LATE-NC was not associated with overall cognitive trajectories. Investigating comorbid pathologies is essential for prognostic stratification and the development of personalized therapeutic strategies in AD.

## 1 Introduction

Alzheimer’s disease (AD) is the leading cause of neurodegenerative dementia and is clinically characterized by progressive memory loss and functional decline. It is pathologically characterized by amyloid plaques and neurofibrillary tangles (Scheltens et al., 2021). However, several clinical subtypes exist, including non-amnestic presentations such as posterior cortical atrophy, logopenic primary progressive aphasia, and frontal variants of AD (Lam et al., 2013). Additionally, various biological and imaging subtypes have been proposed, underscoring the heterogeneity of AD. A recent meta-analysis of AD subtypes suggested that a combination of risk factors, protective factors, and non-AD brain pathologies contributes to differences in typicality and severity (Ferreira et al., 2020). With the advent of anti-amyloid therapy, which is expected to exert a disease-modifying effect (Perneczky et al., 2023), subtyping of AD might be helpful in regard of patient stratification and prediction of therapeutic responses.

Mixed pathology refers to the coexistence of multiple protein aggregation pathologies that may contribute to diverse clinical manifestations, including neurological and psychiatric conditions (Rahimi and Kovacs, 2014). AD-related neuropathological changes (AD-NC), Lewy body-related neuropathological changes (LB-NC), and limbic-predominant age-related TDP-43 encephalopathy-related neuropathological changes (LATE-NC) are among the most frequently observed pathologies in the aging brain (Karanth et al., 2020). Mixed pathology is common in both cognitively normal older individuals and pathology-confirmed AD cases (Devanand et al., 2022; Karanth et al., 2020). However, the impact of coexisting pathologies on clinical and biological manifestations has not been extensively studied until recently. LB-associated biomarkers are known to exert additional detrimental effects on both cross-sectional (Chung et al., 2015) and longitudinal cognitive trajectories in AD (Collij et al., 2024), and vice versa (Biundo et al., 2021). Similarly, patients with mixed LATE-NC and AD-NC exhibit faster cognitive decline than those with either pathology alone (Kapasi et al., 2020). Furthermore, a recent study demonstrated that the coexistence of AD-NC, LB-NC, and LATE-NC—termed quadruple pathology—is common and associated with an aggressive disease course and severe dementia in a community-based cohort (Karanth et al., 2020). However, the impact of these three common pathologies on cognitive trajectories remains inadequately explored.

In this study, we aimed to investigate the impact of LB-NC and LATE-NC on the clinical trajectory of patients with both clinically and pathologically confirmed AD, focusing on cognitive function and neuropsychiatric symptoms. We hypothesized that LB-NC and LATE-NC have distinct impact on the progression of cognitive and neuropsychiatric symptoms in patients with AD.

## 2 Materials and methods

### 2.1 Source of data

The data analyzed in this study were obtained from the Alzheimer’s Disease Neuroimaging Initiative (ADNI) database^1^ and were downloaded on July 14, 2024. The ADNI is a longitudinal study initiated in 2003 as a public-private partnership to evaluate biomarkers associated with the progression of mild cognitive impairment (MCI) and early AD. The inclusion and exclusion criteria are detailed in the ADNI protocol.^2^

### 2.2 Participants and clinical covariates

As of July 2024, autopsy data from 110 subjects were available in the ADNI database. Among these, 85 participants who showed evidence of intermediate-to-high levels of AD-NC were enrolled. All participants underwent assessments for Lewy body disease pathology. However, three participants were excluded due to missing TDP-43-related neuropathological assessments. To analyze longitudinal cognitive and neuropsychiatric trajectories, participants with less than 2 years of follow-up before autopsy were excluded (*n* = 4). Consequently, 77 patients were included in the study. Covariates included dementia diagnosis at baseline, age at baseline, sex, years of education, and apolipoprotein-E ε4 (APOE4) genotype status. The ADNI study was approved by the institutional review boards of all participating institutions, and written informed consent was obtained from all participants or their authorized representatives.

### 2.3 Neuropsychological assessments

Participants in the ADNI underwent standardized clinical assessments throughout the study. Baseline evaluations were conducted at study initiation, followed by the first and second follow-ups every 6 months. Subsequent assessments occurred annually starting 12 months after baseline, with additional evaluations at 18 months for participants with mild cognitive impairment. Detailed study schedules are available online.^3^

**TABLE 1:** Baseline clinical characteristics, and postmortem pathological findings.

	Total group	LB-NC (−)	LB-NC (+)	*p*-value	LATE-NC (−)	LATE-NC (+)	*p*-value
N	**77**	39	38		38	39	
Demography							
Age at death, years	82.8 ± 7.3	84.2 ± 7.1	81.4 ± 7.2	0.140	81.6 ± 6.9	84.1 ± 7.4	0.092
Sex, male, *n* (%)	55 (71%)	25 (64%)	30 (79%)	0.149	27 (71%)	28 (72%)	> 0.9
Education, years	16.3 ± 2.8	15.9 ± 2.6	16.7 ± 2.8	0.173	16.8 ± 2.4	15.7 ± 3.0	0.190
Handedness, right, n (%)	71 (92%)	37 (95%)	34 (89%)	0.431	35 (92%)	36 (92%)	> 0.9
Race, white, *n* (%)	75 (97%)	38 (97%)	37 (97%)	> 0.9	37(97%)	38 (97%)	> 0.9
APOE4 carrier, *n* (%)	56 (73%)	24 (62%)	32 (84%)	0.026	29 (76%)	27 (69%)	0.485
Age at first visit, years	76.1 ± 6.9	77.0 ± 6.6	75.2 ± 7.1	0.401	75.5 ± 7.1	76.7 ± 6.7	0.586
Follow up duration, years	6.3 ± 3.1	6.9 ± 3.0	5.6 ± 3.1	0.021	5.6 ± 3.0	7.0 ± 3.0	0.034
Initial cognitive status, *n* (%)				0.101			0.476
Normal cognition	9 (12%)	7 (18%)	2 (5.3%)		4 (11%)	5 (13%)	
Mild cognitive impairment	35 (45%)	19 (49%)	16 (42%)		20 (53%)	15 (38%)	
Dementia	33 (43%)	13 (33%)	20 (53%)		14 (37%)	19 (49%)	
Baseline dementia *n* (%)	33 (43%)	13 (33%)	20 (53%)	0.087	14 (37%)	19 (49%)	0.292
Baseline cognitive scores							
MMSE	25.71 ± 2.94	26.23 ± 2.88	25.18 ± 2.90	0.136	25.82 ± 2.84	25.62 ± 3.03	0.782
ADAS-Cog total, 13	23.40 ± 8.17	21.18 ± 7.98	25.68 ± 7.72	0.028	22.55 ± 7.75	24.23 ± 8.47	0.338
Clock making test	3.82 ± 1.28	4.08 ± 1.00	3.55 ± 1.46	0.173	3.74 ± 1.16	3.90 ± 1.37	0.315
Clock copying test	4.62 ± 0.70	4.72 ± 0.55	4.53 ± 0.82	0.357	4.61 ± 0.78	4.64 ± 0.62	> 0.9
Verbal fluency test, animal	14.38 ± 5.29	14.38 ± 4.96	14.37 ± 5.61	> 0.9	15.16 ± 5.94	13.62 ± 4.45	0.209
Trail making test part A	52.95 ± 27.59	50.54 ± 24.52	55.42 ± 30.22	0.596	57.68 ± 30.45	48.33 ± 23.58	0.243
Trail making test part B	159.66 ± 83.68	156.39 ± 84.51	163.20 ± 82.63	0.715	171.08 ± 86.39	148.54 ± 79.40	0.339
Boston naming test	25.12 ± 4.19	25.38 ± 3.53	24.84 ± 4.77	> 0.9	25.24 ± 4.38	25.00 ± 3.99	0.538
Rey auditory learning test, delayed recall	8.78 ± 3.85	8.79 ± 4.17	8.76 ± 3.50	> 0.9	9.34 ± 3.72	8.23 ± 3.90	0.230
NPI-Q total score	2.59 ± 3.37	2.03 ± 3.02	3.19 ± 3.66	0.134	3.42 ± 3.87	1.73 ± 2.53	0.028
Pathological findings							
Alzheimer’s disease pathology, *n* (%)				0.142			0.800
High	64 (83%)	30 (77%)	34 (89%)		32 (84%)	32 (82%)	
Intermediate	13 (17%)	9 (23%)	4 (11%)		6 (16%)	7 (18%)	
Thal stage				0.053			0.824
1	0	0	0		0	0	
2	0	0	0		0	0	
3	5	5	0		3	2	
4	20	11	9		9	11	
5	52	23	29		26	26	
Braak stage				0.136			0.519
1	0	0	0		0	0	
2	0	0	0		0	0	
3	5	5	0		3	2	
4	7	4	3		2	5	
5	44	20	24		24	20	
6	21	10	11		9	12	
CERAD stage				0.144			0.272
0	3	3	0		3	0	
1	6	3	3		2	4	
2	11	3	8		6	5	
3	57	30	27		27	30	
TDP-43 pathology, *n* (%)	39 (51%)	20 (51%)	19 (50%)	> 0.9			NA
Lewy body disease pathology, *n* (%)				NA			0.247
None	39 (51%)	39 (100%)	0 (0%)		19 (50%)	20 (51%)	
Olfactory bulb	2 (2.6%)		2 (5.3%)		0 (0%)	2 (5.1%)	
Amygdala predominant	13 (17%)		13 (34%)		9 (24%)	4 (10%)	
Brainstem predominant	2 (2.6%)		2 (5.3%)		2 (5.3%)	0 (0%)	
Limbic (transitional)	8 (10%)		8 (21%)		3 (7.9%)	5 (13%)	
Neocortical (diffuse)	13 (17%)		13 (34%)		5 (13%)	8 (21%)	
Vascular pathology							
CAA pathology				0.307			0.762
None	5	4	1		2	3	
Mild	36	20	16		20	16	
Moderate	19	7	12		8	11	
Severe	17	8	9		8	9	
Infarction (macro/micro/none)				0.898			0.388
None	55	28	27		27	28	
Microscopic infarction	15	8	7		9	6	
Gross infarction	7	3	4		2	5	
Hemorrhage (macro/micro/none)				0.184			0.600
None	71	35	36		34	37	
Microbleed	0	0	0		0	0	
Gross hemorrhage	5	4	1		3	2	
Atherosclerosis				0.757			0.220
None	26	13	13		10	16	
Mild	34	16	18		18	16	
Moderate	13	7	6		9	4	
Severe	4	3	1		1	3	
Arteriolosclerosis				0.852 0.227			0.617 0.562
None	4	2	2		1	3	
Mild	44	21	23		23	21	
Moderate	23	12	11		12	11	
Severe	6	4	2		2	4	
White matter rarefaction	12	8	4		5	7	

Data are expressed as mean ± standard deviation or number (percentage). Group comparisons were performed using Wilcoxon rank sum exact test; Pearson’s Chi-squared test; Wilcoxon rank sum test; Fisher’s exact test as appropriate. ADAS-cog 13, Alzheimer’s Disease Assessment Scale-Cognitive subscale 13; APOE4, apolipoprotein E ε4; LATE-NC, limbic-predominant age-related TDP-43 encephalopathy-related neuropathological changes; LB-NC, Lewy body-related neuropathological changes; MMSE, mini-mental status examination; NPI-Q, neuropsychiatric inventory questionnaire.

Global cognition was assessed using the Mini-Mental State Examination (MMSE) and the Alzheimer’s Disease Assessment Scale-Cognitive Subscale 13 (ADAS-Cog 13) total scores. Specific neuropsychological tests included the clock drawing test (CDT), delayed recall of the Rey auditory verbal learning test (RAVLT), Boston naming test (BNT), animal category fluency test (CFT-animal), and the trail making test (TMT) parts A and B. These assessments were administered across the ADNI-1 to ADNI-4 cohorts. For longitudinal comparisons, *z*-scores were derived based on participants with normal cognition at baseline in the ADNI database (*n* = 897) for each cognitive assessment.

Caregivers completed the Neuropsychiatric Inventory Questionnaire (NPI-Q), which consists of 12 behavioral and psychological symptoms, including delusions, hallucinations, agitation, depression, anxiety, euphoria, apathy, disinhibition, irritability, aberrant motor behavior, sleep disturbances, and appetite changes (Cummings et al., 1994). Severity was rated on a Likert scale, yielding a maximum total score of 36. NPI-Q total and subdomain scores were collected throughout follow-up.

### 2.4 Neuropathological assessments

For study participants, the mean postmortem interval from death to the initiation of assessments was 13.21 ± 12.30 h. Neuropathological evaluations adhered to the National Institute on Aging-Alzheimer’s Association (NIA-AA) guidelines (Hyman et al., 2012). Detailed protocols are available in the ADNI database.^1^ AD-NC levels were determined using the Thal phase for Aβ plaques, the Braak stage for neurofibrillary tangles or tau burden, and the Consortium to Establish a Registry for AD (CERAD) score for neuritic plaques, in line with consensus criteria (Cairns et al., 2010). Cases were classified as having low, intermediate, or high AD-NC, with intermediate-to-high levels indicative of AD. Due to limited sample size, Lewy body subtypes were not stratified but combined into a single LB-NC group. Cases were classified as LB-NC-positive if Lewy bodies were identified in the olfactory bulb, brainstem, limbic system, amygdala, or neocortex (Chung et al., 2015). Similarly, the presence of LATE-NC was determined based on TDP-43 proteinopathy in the amygdala, hippocampus, entorhinal cortex, or inferior temporal cortex (Nelson et al., 2019). Semiquantitative burden and distribution data were available for LB-NC, LATE-NC, and vascular pathologies. However, as stratification of each neuropathology would have substantially fragmented the cohort, the primary analyses used binary classifications based on the presence or absence of LB-NC and LATE-NC. Vascular pathologies, including infarction, hemorrhage, atherosclerosis, arteriolosclerosis, cerebral amyloid angiopathy (CAA), and white matter rarefaction, were investigated using semiquantitative scoring using none, mild, moderate, or severe (Perrin et al., 2024).

### 2.5 Statistical analysis

Statistical analyses were conducted using R (version 4.4.1) and SPSS (version 26.0; IBM Corporation, Armonk, NY). Baseline age and age at death, sex, years of education, handedness, race, APOE4 status, follow-up duration, and neuropathological findings for AD-NC (Thal phase, Braak stage, and CERAD score) and vascular pathologies were compared based on the presence of LB- and LATE-NC. Independent *t*-tests and chi-square tests were applied to compare continuous and categorical variables, respectively. The impact of LB- and LATE-NC on baseline cognition and neuropsychiatric symptoms was analyzed using multivariate linear regression, with baseline age, sex, education, APOE4 status, and baseline dementia diagnosis as covariates. Linear mixed models were used to evaluate the effects of LB- and LATE-NC on longitudinal cognitive scores and neuropsychiatric burden. Dependent variables included standardized *z*-scores for the MMSE, ADAS-Cog 13, and individual neuropsychological tests, as well as raw NPI-Q total scores in separate models. Baseline age, sex, education, APOE4 status, and baseline dementia were fixed effects, while participants were included as random effects. Interaction terms (e.g., LB-NC × time and LATE-NC × time) were tested to assess the effects of LB- and LATE-NC over time. The effect of interaction between LB-NC and LATE-NC over time (LB-NC × LATE-NC × time) was tested, and retained in the model only if statistically significant. As interaction among LB-NC, LATE-NC, and time was not significant for all clinical trajectories, this interaction term was not included in the final statistical model. False discovery rate correction was applied for multiple comparisons, with a *p*-value and corrected *Q*-value < 0.05 considered statistically meaningful.

## 3 Results

### 3.1 Baseline characteristics

The demographic, clinical, and neuropathological characteristics of the study participants are summarized in Table 1. Of the 77 participants, 55 (71%) were male participants, with a mean age at death of 82.8 years. The mean baseline age was 76.1 years, and 33 (43%) patients had dementia at baseline. Fifty-six participants (73%) were APOE4 carriers, and the mean follow-up duration was 6.3 ± 3.1 years. Among the 77 participants, 38 (49.4%) had LB-NC, and 39 (50.6%) had LATE-NC. Nineteen participants (24.7%) had both LB-NC and LATE-NC. Among 38 participants with LB-NC, 2 had olfactory bulb-predominant, 13 had amygdala-predominant, 2 had brainstem-predominant, 8 had limbic, and 13 had neocortical LB-NC. No significant association was observed between the presence of LB-NC and LATE-NC. Participants with LB-NC had a higher proportion of APOE4 carriers, a shorter follow-up duration, and higher ADAS-Cog 13 scores than those without LB-NC. Other demographic and clinical measures were comparable between participants with and without LB-NC. Participants with LATE-NC had longer duration of follow-up and lower baseline NPI-Q scores, while other demographic and clinical measures were comparable between those with and without LATE-NC. The distributions of Thal phase, Braak stage, CERAD score were comparable according to LB-NC and LATE-NC status. Vascular pathologies, including infarction, hemorrhage, atherosclerosis, arteriolosclerosis, CAA, and white matter rarefaction, were also comparable between groups.

### 3.2 Baseline association between neuropathological findings and clinical measures

The cross-sectional impact of LB-NC and LATE-NC on baseline cognitive test scores are summarized in Table 2. The presence of the APOE4 allele was associated with poorer RAVLT performance (β = −1.37, SE = 0.39, *p* < 0.001, *Q* < 0.001), and baseline dementia was associated with worse cognitive performance across all neuropsychological tests. The presence of LB-NC was not associated with any baseline cognitive test scores after adjusting for covariates. However, the presence of LATE-NC was associated with better TMT-A and TMT-B performance, independent of covariates. The associations between LB-NC, LATE-NC, and neuropsychiatric burden are summarized in Table 3. The presence of the APOE4 allele, baseline dementia, and the presence of LB-NC were not associated with any neuropsychiatric symptoms. However, the presence of LATE-NC was associated with lower baseline sleep disturbance (β = −0.37, SE = 0.13, *p* = 0.006, *Q* = 0.026).

**TABLE 2:** Cross-sectional effect of LB-NC and LATE-NC on baseline cognition.

	APOE4 carrier			Baseline dementia			LB-NC			LATE-NC		
	β (SE)	*p*	*q*	β (SE)	*p*	*q*	β (SE)	*p*	*q*	β (SE)	*p*	*q*
MMSE	−0.75 (0.50)	0.139	0.277	−3.97 (0.45)	< 0.001	**< 0.001**	−0.07 (0.46)	0.873	0.873	0.27 (0.44)	0.545	0.727
ADAS-Cog 13	−0.67 (0.42)	0.109	0.218	−2.20 (0.37)	< 0.001	**< 0.001**	−0.43 (0.38)	0.261	0.348	−0.19 (0.36)	0.609	0.609
Clock drawing	−0.95 (0.49)	0.054	0.108	−2.16 (0.44)	< 0.001	**< 0.001**	−0.16 (0.44)	0.72	0.718	0.41 (0.42)	0.333	0.444
Clock copying	−0.60 (0.37)	0.106	0.213	−0.97 (0.33)	0.004	**0.018**	−0.07 (0.34)	0.84	0.844	0.26 (0.32)	0.431	0.574
BNT (76)	0.33 (0.48)	0.497	0.497	−1.49 (0.44)	0.001	**0.004**	−0.34 (0.44)	0.449	0.497	0.29 (0.42)	0.494	0.497
RAVLT, delayed recall	−1.37 (0.39)	< 0.001	**0.001**	−1.46 (0.35)	< 0.001	**<0.001**	0.60 (0.35)	0.10	0.127	−0.42 (0.34)	0.218	0.218
CFT animal	−0.08 (0.24)	0.755	0.829	−0.87 (0.21)	< 0.001	**< 0.001**	0.11 (0.22)	0.621	0.829	−0.05 (0.21)	0.829	0.829
TMT-A	1.05 (0.55)	0.058	0.078	−2.52 (0.49)	< 0.001	**< 0.001**	−0.23 (0.50)	0.653	0.653	1.41 (0.48)	0.004	**0.008**
TMT-B	0.67 (0.49)	0.175	0.233	−2.43 (0.45)	< 0.001	**< 0.001**	0.04 (0.45)	0.924	0.924	1.09 (0.43)	0.013	**0.027**

Data are the results of multivariate linear regression models for standardized *z*-scores of baseline tests as dependent variables after controlling baseline age, sex, years of education as covariates. The predictors are APOE4 carrier, baseline dementia, LB-NC and LATE-NC. The *p*-values were corrected for multiple comparisons using the false discovery rate method. ADAS-Cog 13, Alzheimer’s Disease Assessment Scale-Cognitive subscale 13; APOE4, apolipoprotein E ε4; BNT, Boston naming test; CFT, confrontation naming test; LATE-NC, limbic-predominant age-related TDP-43 encephalopathy-related neuropathological changes; LB-NC, Lewy body-related neuropathological changes; MMSE, mini-mental status examination; RAVLT, Rey auditory verbal learning test; TMT-A, Trail Making Test Part A; TMT-B, Trail Making Test Part B. Bold values indicate significance after correction for multiple comparisons.

**TABLE 3:** Impact of LB-NC and LATE-NC on baseline neuropsychiatric symptoms.

	APOE4 carrier			Baseline dementia			LB-NC			LATE-NC		
	β (SE)	*p*	*q*	β (SE)	*p*	*q*	β (SE)	*p*	*q*	β (SE)	*p*	*q*
Total NPI-Q score	0.07 (0.89)	0.934	0.934	1.77 (0.81)	0.032	0.063	0.80 (0.82)	0.328	0.437	−1.85 (0.78)	0.021	0.063
Delusion	0.13 (0.14)	0.333	0.443	0.16 (0.13)	0.220	0.440	−0.01 (0.13)	0.919	0.919	−0.23 (0.12)	0.073	0.291
Hallucination	0.07 (0.08)	0.427	0.570	0.12 (0.07)	0.117	0.295	−0.11 (0.08)	0.147	0.295	−0.03 (0.07)	0.657	0.657
Agitation/aggression	−0.08 (0.18)	0.675	0.675	0.12 (0.16)	0.477	0.636	0.12 (0.17)	0.460	0.636	−0.12 (0.16)	0.441	0.636
Depression/dysphoria	−0.03 (0.16)	0.841	0.841	0.10 (0.14)	0.475	0.841	0.07 (0.14)	0.639	0.841	−0.13 (0.14)	0.336	0.841
Anxiety	−0.17 (0.15)	0.251	0.334	0.23 (0.13)	0.090	0.179	0.09 (0.14)	0.499	0.499	−0.22 (0.13)	0.087	0.179
Elation/euphoria	0.01 (0.04)	0.821	0.821	−0.05 (0.04)	0.242	0.355	0.05 (0.04)	0.220	0.355	−0.04 (0.04)	0.266	0.355
Apathy/indifference	−0.17 (0.17)	0.321	0.543	0.09 (0.15)	0.543	0.543	0.13 (0.15)	0.411	0.543	−0.10 (0.15)	0.483	0.543
Disinhibition	0.13 (0.11)	0.248	0.642	0.10 (0.10)	0.321	0.642	−0.04 (0.10)	0.696	0.773	−0.03 (0.10)	0.773	0.773
Irritability/lability	0.20 (0.18)	0.274	0.366	0.31 (0.16)	0.057	0.156	0.14 (0.16)	0.382	0.382	−0.28 (0.16)	0.078	0.156
Aberrant motor behavior	0.01 (0.12)	0.965	0.965	0.10 (0.11)	0.368	0.490	0.10 (0.11)	0.352	0.490	−0.22 (0.11)	0.040	0.160
Sleep	0.12 (0.15)	0.443	0.443	0.31 (0.14)	**0.025**	0.050	0.18 (0.14)	0.192	0.256	−0.37 (0.13)	0.006	**0.026**
Appetite	−0.18 (0.13)	0.157	0.433	0.09 (0.11)	0.432	0.577	0.02 (0.12)	0.889	0.889	−0.14 (0.11)	0.217	0.433

Data are the results of multivariate linear regression models for baseline neuropsychiatric inventory scores as dependent variables after controlling baseline age, sex, years of education as covariates. The predictors are APOE4 carrier, baseline dementia, LB-NC and LATE-NC. The *p*-values were corrected for multiple comparisons using the false discovery rate method. APOE4, apolipoprotein E ε4; LATE-NC, limbic-predominant age-related TDP-43 encephalopathy-related neuropathological changes; LB-NC, Lewy body-related neuropathological changes; NPI-Q, neuropsychiatric inventory questionnaire. Bold values indicate significance after correction for multiple comparisons.

### 3.3 Impact of LB-NC and LATE-NC on longitudinal cognitive trajectory

**FIGURE 1:**
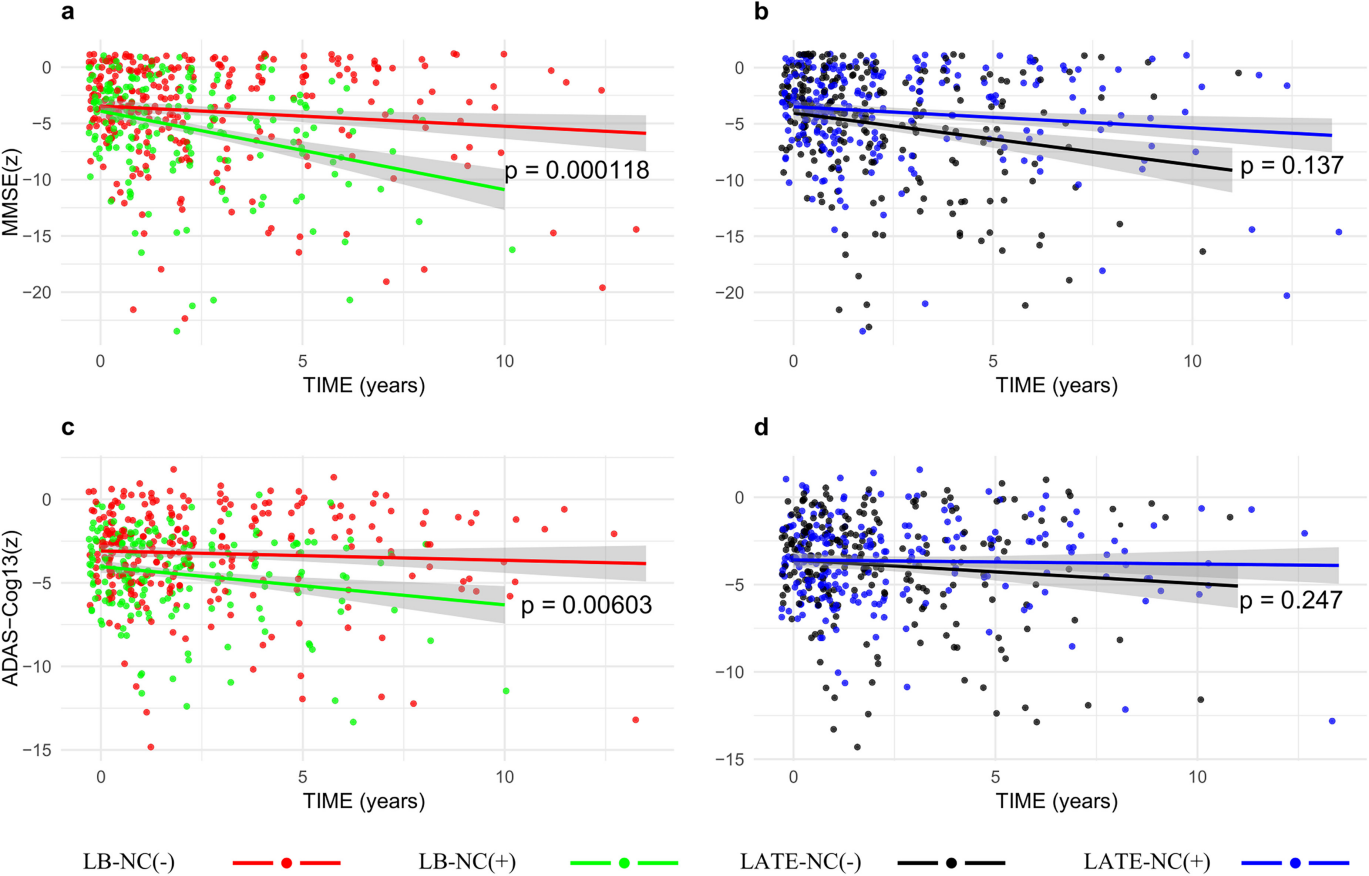
Longitudinal decline of global cognition according to the presence of Lewy body- (LB-NC) and limbic-predominant age-related TDP-43 encephalopathy (LATE) related neuropathological changes (LATE-NC). Patients with LB-NC (green line) showed a faster decline in Mini-Mental State examination (MMSE) **(A)** (*p* < 0.001) and Alzheimer’s Disease Assessment Scale-Cognitive subscale 13 (ADAS-Cog 13) **(B)** (*p* = 0.006) scores than those without LB-NC (red line). Patients with LATE-NC (blue line) and those without LATE-NC (black line) had comparable declines in MMSE **(C)** (*p* = 0.137) and ADAS-Cog 13 **(D)** (*p* = 0.247) scores.

**FIGURE 2:**
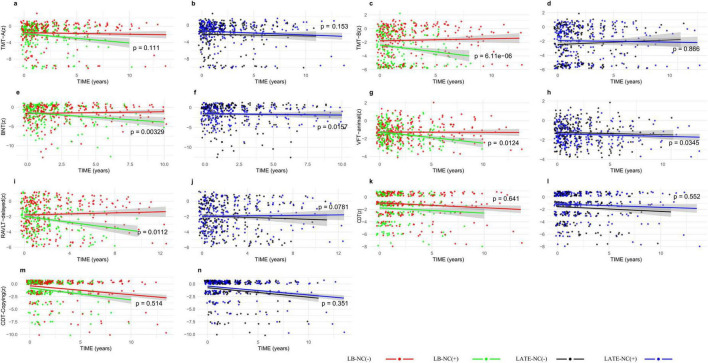
Longitudinal decline of specific neuropsychological test scores according to the presence of Lewy body- (LB-NC) and limbic-predominant age-related TDP-43 encephalopathy (LATE) related neuropathological changes (LATE-NC). Patients with LB-NC (green line) showed faster declines in the Boston naming test (BNT), delayed recall of Rey auditory learning test (RAVLT), animal category fluency test (CFT-animal), and trail making test - part B (TMT-B) than those without LB-NC (red line). Patients with (blue line) and without (black line) LATE-NC showed a comparable decline in neuropsychological tests. Panels **(a,c,e,g,i,k,m)** depict the trajectories of TMT-A, TMT-B, BNT, VFT-animal, RAVLT delayed recall, CDT, and CDT copying by LB-NC status, respectively; panels **(b,d,f,h,j,l,n)** depict the trajectories of the same tests by LATE-NC status, respectively.

The longitudinal effects of LB-NC and LATE-NC on cognitive scores were analyzed using LMMs, including LB-NC × time and LATE-NC × time interaction terms (Table 4 and Figures 1, 2). The three-way interaction among LB-NC, LATE-NC, and time (LB-NC × LATE-NC × time) was not significant for any cognitive outcome, and was therefore removed from the statistical models (Supplementary Table S2). All cognitive test scores declined over time. The presence of LB-NC or LATE-NC was not associated with cognitive scores during follow-up. However, the presence of LB-NC was associated with a faster decline in MMSE (β = −0.54, SE = 0.14, *p* < 0.001, *Q* < 0.001) and ADAS-Cog 13 (β = −0.22, SE = 0.08, *p* = 0.006, *Q* = 0.022). Specifically, LB-NC was associated with a more rapid decline in BNT (β = −0.24, SE = 0.08, *p* = 0.003, *Q* = 0.012), RAVLT (β = −0.15, SE = 0.06, *p* = 0.011, *Q* = 0.041), CFT-animal (β = −0.08, SE = 0.03, *p* = 0.012, *Q* = 0.045), and TMT-B (β = −0.35, SE = 0.08, *p* < 0.001, *Q* < 0.001). In contrast, LATE-NC was associated with a slower decline in BNT (β = 0.18, SE = 0.07, *p* = 0.016, *Q* = 0.043).

**TABLE 4:** Longitudinal effect of LB-NC and LATE-NC on cognitive function.

	Time (years)			LB-NC			LB-NC × time			LATE-NC			LATE-NC × time		
	β (SE)	*p*	*q*	β (SE)	*p*	*q*	β (SE)	*p*	*q*	β (SE)	*p*	*q*	β (SE)	*p*	*q*
MMSE (77)	−0.85 (0.11)	< 0.001	**< 0.001**	0.73 (0.81)	0.372	0.508	−0.54 (0.14)	< 0.001	**< 0.001**	0.64 (0.78)	0.415	0.508	0.19 (0.13)	0.137	0.300
ADAS-Cog 13 (77)	−0.47 (0.06)	< 0.001	**< 0.001**	−0.12 (0.51)	0.812	0.957	−0.22 (0.08)	0.006	**0.022**	−0.01 (0.48)	0.982	0.982	0.08 (0.07)	0.247	0.453
Clock drawing (77)	−0.27 (0.06)	< 0.001	**< 0.001**	−0.29 (0.42)	0.482	0.653	−0.04 (0.08)	0.641	0.653	0.31 (0.40)	0.437	0.653	0.04 (0.07)	0.552	0.653
Clock copying (77)	−0.39 (0.07)	< 0.001	**< 0.001**	0.07 (0.41)	0.863	0.946	−0.06 (0.09)	0.514	0.706	0.34 (0.39)	0.389	0.701	0.08 (0.08)	0.351	0.701
BNT (76)	−0.33 (0.06)	< 0.001	**< 0.001**	0.20 (0.52)	0.708	0.714	−0.24 (0.08)	0.003	**0.012**	0.42 (0.50)	0.399	0.548	0.18 (0.07)	0.016	0.043
RAVLT, delayed recall (77)	−0.11 (0.05)	0.024	0.067	0.37 (0.37)	0.314	0.493	−0.15 (0.06)	0.011	**0.041**	−0.03 (0.35)	0.934	0.934	−0.09 (0.05)	0.078	0.172
CFT animal (77)	−0.16 (0.03)	< 0.001	**< 0.001**	0.18 (0.22)	0.414	0.570	−0.08 (0.03)	0.012	**0.045**	−0.02 (0.21)	0.931	0.931	0.06 (0.03)	0.035	0.095
TMT-A (77)	−0.40 (0.09)	< 0.001	**< 0.001**	−0.06 (0.59)	0.913	0.913	−0.17 (0.11)	0.111	0.304	1.08 (0.56)	0.056	0.206	0.14 (0.10)	0.153	0.336
TMT-B (74)	−0.19 (0.06)	0.002	**0.007**	−0.06 (0.43)	0.883	0.883	−0.35 (0.08)	< 0.001	**< 0.001**	0.83 (0.41)	0.046	0.128	−0.01 (0.07)	0.866	0.883

The data in the table represent the results of linear mixed-effect models for standardized *z*-scores of each cognitive test at each time point, controlling for APOE4 carrier status, baseline dementia, baseline age, sex, and years of education as covariates. The primary predictors included the presence of LBD and LATE pathologies and their interaction terms with time. Results are presented with the estimate (β), standard error (SE), *p*-value, and *q*-value, corrected by the false discovery rate method. The total sample size (*N*) was calculated for each variable. The presence of LB-NC was associated with a faster decline in MMSE, ADAS-Cog13, BNT, RAVLT, and CFT scores, whereas the presence of LATE-NC was associated with a slower decline in BNT scores. ADAS-cog 13, Alzheimer’s Disease Assessment Scale-Cognitive subscale 13; APOE4, apolipoprotein E ε4; BNT, Boston naming test; CFT, confrontation naming test; LATE-NC, limbic-predominant age-related TDP-43 encephalopathy-related neuropathological changes; LB-NC, Lewy body-related neuropathological changes; MMSE, mini-mental status examination; RAVLT, Rey auditory verbal learning test; TMT-A, Trail Making Test Part A; TMT-B, Trail Making Test Part B. Bold values indicate significance after correction for multiple comparisons.

### 3.4 Impact of LB-NC and LATE-NC on longitudinal neuropsychiatric trajectory

The longitudinal effects of LB-NC and LATE-NC on neuropsychiatric symptoms are summarized in Supplementary Table S1. The three-way interaction among LB-NC, LATE-NC, and time (LB-NC × LATE-NC × time) was not significant for any neuropsychiatric symptoms, and therefore was removed from the statistical models (Supplementary Table S3). Neither LB-NC nor LATE-NC were associated with neuropsychiatric symptoms or overall neuropsychiatric burden over time. However, time was positively associated with total NPI-Q score (β = 0.47, SE = 0.13, *p* < 0.001, *Q* = 0.003), apathy (β = 0.09, SE = 0.03, *p* = 0.001, *Q* = 0.015), and sleep disturbance (β = 0.09, SE = 0.03, *p* = 0.003, *Q* = 0.031).

## 4 Discussion

In this study, we investigated the effects of LB-NC and LATE-NC on cognitive decline and neuropsychiatric burden in patients with pathologically confirmed AD. The major findings were as follows: (1) the presence of LB-NC and LATE-NC was common; (2) the presence of LB-NC exacerbated global and specific cognitive decline, independent of the natural course of AD-NC. However, the presence of LATE-NC was not associated with faster cognitive deterioration; and (3) the presence of LB-NC and LATE-NC was not associated with longitudinal neuropsychiatric burden, whereas overall participants exhibited a time-dependent increase in total neuropsychiatric burden, particularly in apathy and sleep disturbances. Our study has several strengths. First, participants underwent frequent, scheduled longitudinal follow-up over a mean duration of 6 years, allowing robust characterization of clinical trajectories. Second, all participants had neuropathologically confirmed diagnoses, which are gold standard for the disease classification. Third, we investigated AD-NC, LB-NC, and LATE-NC within the same statistical models, thereby minimizing potential confounding across coexisting neuropathologies. Together, these strengths provide a plausible framework for understanding the clinical heterogeneity of AD.

In the era of anti-amyloid therapy, clinical and neuropathological heterogeneity in AD remains crucial for patient stratification and therapeutic decision-making (Duara and Barker, 2022). Mixed pathology may contribute to phenotypic heterogeneity and clinical progression in AD, as well as to the response to AD-NC-targeted therapeutic strategies. Mixed pathology is highly prevalent in clinically defined AD, and LB-NC and LATE-NC are neurodegenerative pathologies commonly observed in AD (Spina et al., 2021) and aging brains (Rahimi and Kovacs, 2014). Vascular neuropathologies are also commonly observed in the individuals with cognitive impairment (Oveisgharan et al., 2022). However, the prevalence of vascular pathologies was comparable according to LB-NC and LATE-NC status, and a significant burden of vascular pathology was not common in this cohort, suggesting that confounding by vascular pathology is likely to have been limited in the present study. The prevalence of LB-NC and LATE-NC in patients with AD has been reported up to 39 and 57%, respectively (Meneses et al., 2021; Spina et al., 2021). In this study, 39 (51%) of 77 patients had LB-NC, and 39 (51%) had LATE-NC, which is comparable to previous neuropathological studies, ascertaining that both neuropathologic changes are common in pathologically confirmed AD. However, the association between LB-NC and LATE-NC has not been thoroughly investigated. Some studies have demonstrated a possible association between LB-NC and LATE-NC (Agrawal et al., 2021). In contrast, we did not observe a statistically significant association between LB-NC and LATE-NC in our cohort. As our sample size was modest and prior studies have reported heterogeneous results, this finding should not be interpreted as definitive evidence of biological independence between LB-NC and LATE-NC. Among the subtypes of LB-related disorders, dementia with Lewy bodies (DLB) with coexisting AD had a higher prevalence of TDP-43 pathology than pure DLB, Parkinson’s disease dementia, or Parkinson’s disease (Nakashima-Yasuda et al., 2007). Considering the potential interaction between AD-NC and LATE-NC (Meneses et al., 2021), these associations may be attributed to comorbid AD pathology. In addition, olfactory bulb- and amygdala-predominant LB pathology may influence clinical phenotype differently from brainstem, limbic, or neocortical LB pathology. In our cohort, 15 LB-NC positive cases were classified as olfactory bulb- or amygdala-predominant. When these cases were excluded, the remaining sample size was substantially reduced, limiting statistical power and attenuating statistical significance (data not shown). However, due to the reduced sample size, we could not determine whether olfactory bulb- or amygdala-predominant LB-NC exerts effects distinct from those of other anatomical distributions of LB-NC. Therefore, larger community-based neuropathological studies are warranted to clarify the associations among AD-NC, LB-NC, and LATE-NC, and to determine whether the clinical effect of LB-NC differs according to its anatomical distribution.

The presence of LB-NC was not associated with cognitive manifestations at baseline. However, it was associated with faster deterioration in both global and specific cognitive functions, including memory, attention, and executive function. The impact of LB-NC on cognitive function has been investigated in both cross-sectional and longitudinal studies. A previous study demonstrated that the presence of LB-NC is associated with LB-specific clinical features, including sleep disturbances and hallucinations (Devanand et al., 2022), as well as cognitive impairments such as deficits in global cognition and memory (Palmqvist et al., 2023; Quadalti et al., 2023). Moreover, a recent study revealed that patients with a positive cerebrospinal fluid alpha-synuclein seed amplification assay exhibited faster cognitive decline (Quadalti et al., 2023). These findings are in line with the findings in our study, indicating that LB-NC has an additive deteriorative effect on global cognition and specific cognitive functions. In contrast, in our study, the presence of LATE-NC was not associated with worse cognitive function at baseline or over time. Rather, it was associated with better executive function at baseline and a slower decline in language function. Recent studies have shown that LATE-NC is associated with dementia, independent of other neurodegenerative pathologies (Mikhailenko et al., 2025), and with impairments in global cognition and episodic memory (Nag et al., 2017). In a large-scale community cohort, comorbid LATE-NC with AD-NC was associated with faster declines in MMSE scores compared to AD-NC alone. However, pure LATE-NC was linked to a slower cognitive decline compared to pure AD-NC (Kapasi et al., 2020). Previous studies, however, did not employ scheduled follow-ups for cognitive testing and most studies investigated retrospective, population-based cohort data. Moreover, these studies varied in the proportion of the presence of LB-NC, and their longitudinal models for cognitive decline did not include the impact of comorbid pathologies, including LB-NC. In our study, patients with LATE-NC had a longer follow-up duration, which might be attributed to slower cognitive and functional decline. Another possible explanation is that the LATE-NC detected at autopsy may have developed later during follow-up period, and therefore may have had less opportunity to exert its effect on the longitudinal clinical trajectories. As LATE-NC cannot currently be detected reliably in vivo, autopsy-based studies cannot determine when TDP-43 pathology emerged during the disease course or when it began to exert clinically meaningful effects. Accordingly, future studies incorporating longitudinal *in vivo* biomarkers for TDP-43 will be necessary to clarify its temporal contribution to clinical progression. A recent neuropathological study revealed that the presence of LATE-NC was associated with worse cognition and a faster decline in MMSE scores among participants with LBD. However, LATE-NC was not associated with cognitive function or decline in participants with AD or LBD + AD (Uemura et al., 2022). Therefore, the impact of LATE-NC on cognitive function in patients with AD might stem from interactions between LATE-NC and LB-NC in previous studies. As interactions between multiple neuropathologies may be complex, further large-scale, well-defined subpopulation studies are warranted. Additionally, given that the prevalence of LATE-NC is particularly high in the oldest-old population (Wang et al., 2022) and cognitive reserve diminishes with age, the longer follow-up duration might have contributed to the observed cognitive trajectory in participants with LATE-NC in the previous studies. With the recent introduction of anti-amyloid antibody therapy, prognostic stratification of patients with AD is critical. These findings contribute to understanding the variability in clinical progression associated with additional neuropathology in patients with pathologically confirmed AD.

In addition to cognitive impairments, various neuropsychiatric symptoms occur in the AD spectrum and significantly contribute to caregiver burden (Eikelboom et al., 2021). In our study, total neuropsychiatric burden increased over time, with apathy and sleep disturbances worsening progressively in overall participants. Neuropsychiatric symptoms fluctuate throughout the disease course. A previous study showed that most patients exhibit a stable trajectory of neuropsychiatric symptoms (Vik-Mo et al., 2018). Similarly, our results indicate that most neuropsychiatric symptoms did not change over time; however, apathy and sleep disturbances progressively increased until death. At baseline, LB-NC was not associated with specific neuropsychiatric symptoms or overall neuropsychiatric burden, whereas LATE-NC was associated with fewer sleep disturbances. Neither LB-NC nor LATE-NC influenced longitudinal changes in neuropsychiatric symptoms in our study. The presence of LB-NC has been linked to various neuropsychiatric symptoms, including hallucinations, delusions, and depression (Gibson et al., 2023). Additionally, a recent neuropathological study found that patients with LATE-NC exhibited a lower prevalence of anxiety (Liu et al., 2020). However, as neuropsychiatric burden varies over the disease course, the impact of neuropathology on neuropsychiatric symptoms may not be uniform over time. Interestingly, while most neuropsychiatric symptoms remained stable, apathy and sleep disturbances increased over time independently of comorbid LB-NC or LATE-NC. These findings highlight the importance of addressing apathy and sleep disturbances in the management of patients with AD.

Our study had some limitations. First, ADNI is a highly selected prospective research cohort which is predominantly composed of Caucasian ethnicity, which may limit the racial and ethnic generalizability. Furthermore, the relatively small sample size may further limit the generalizability of the findings. However, given that the frequencies of LB-NC and LATE-NC were concordant with prior community-based neuropathological studies, our sample may be representative of the general cognitively impaired population. Second, although this study demonstrated distinct effects of comorbid neuropathologies on AD, the underlying mechanisms driving longitudinal progression remain unclear. A recent study reported an association among LB pathology, occipital hypometabolism, and exacerbation of cognitive decline in AD (Collij et al., 2024). However, the impact of LATE-NC on cognitive decline in both the general population and patients with dementia remains uncertain. Since LB-NC and LATE-NC may influence the clinical trajectory of AD, further studies investigating impact of both neuropathology on clinical trajectory are warranted. Third, although semiquantitative burden and staging data were available, our primary analyses dichotomized LB-NC and LATE-NC because further stratification would have substantially fragmented the cohort and reduced statistical power. Therefore, stage-dependent effects of additional co-pathologies in AD remain to be clarified in larger neuropathological datasets. Fourth, vascular pathology was not included as a covariate in the statistical models. However, the overall burden of vascular pathology was low, and did not differ significantly according to LB-NC or LATE-NC status, rendering substantial confounding by vascular pathology less likely in this cohort. Fifth, our study divided participants using data obtained from autopsy. Considering relatively long follow-up period, classification based on autopsy data may not accurately reflect neuropathological status at baseline. As several fluid and imaging biomarkers for various neuropathologies are being available in these days, future studies should explore pathology-specific biomarkers in larger and more diverse cohorts.

## 5 Conclusion

Our study revealed that comorbid LB-NC was associated with faster cognitive decline, whereas comorbid LATE-NC was associated with slower cognitive decline in patients with pathologically confirmed AD. However, neither LB-NC nor LATE-NC influenced the clinical trajectory of neuropsychiatric symptoms. These findings underscore the importance of considering comorbid neuropathology when stratifying patients with AD.

## Data Availability

The raw data supporting the conclusions of this article will be made available by the authors, without undue reservation.

## Footnotes

1
http://adni.loni.usc.edu

2
http://adni.loni.usc.edu/methods/documents/

3
https://adni.loni.usc.edu/wp-content/uploads/2012/10/adni-complete-study-schedule.pdf

## The Alzheimer’s Disease Neuroimaging Initiative

Data used in the preparation of this article were obtained from the Alzheimer’s Disease Neuroimaging Initiative (ADNI) database (adni.loni.usc.edu). The investigators of the ADNI contributed to the design and implementation of the ADNI and/or provided data, but they did not participate in the analysis or writing of this report. A complete list of the ADNI investigators can be found at http://adni.loni.usc.edu/wpcontent/uploads/how_to_apply/ADNI_Acknowledgement_List.pdf
