# Supplementary material for: Impact of Lewy body and limbic-predominant TDP-43 neuropathology on cognitive and neuropsychiatric trajectory in Alzheimer’s disease: a retrospective neuropathological study
Source: Front Aging Neurosci. 2026 Apr 17;18:1724274. doi: 10.3389/fnagi.2026.1724274 (PMC13132858; doi:10.3389/fnagi.2026.1724274)
Supplement: Supplementary file 1 [file Table_1.docx]

Supplementary Table 1. Longitudinal effect of LB-NC and LATE-NC on neuropsychiatric symptoms

|  | Time (years) | | | LB-NC | | | LB-NC * Time | | | LATE-NC | | | LATE-NC * Time | | |
| --- | --- | --- | --- | --- | --- | --- | --- | --- | --- | --- | --- | --- | --- | --- | --- |
|  | β (SE) | p | q | β (SE) | p | q | β (SE) | p | q | β (SE) | p | q | β (SE) | p | q |
| NPIQ total | 0.47 (0.13) | < 0.001 | 0.003 | 0.40 (0.83) | 0.632 | 0.773 | 0.03 (0.16) | 0.854 | 0.854 | -1.16 (0.78) | 0.142 | 0.522 | -0.17 (0.15) | 0.268 | 0.737 |
| Delusion | 0.01 (0.01) | 0.714 | 0.794 | 0.07 (0.09) | 0.412 | 0.578 | -0.01 (0.02) | 0.722 | 0.794 | -0.13 (0.08) | 0.120 | 0.578 | 0.03 (0.02) | 0.070 | 0.578 |
| Hallucination | -0.01 (0.01) | 0.646 | 0.718 | -0.08 (0.05) | 0.124 | 0.340 | 0.02 (0.02) | 0.172 | 0.378 | -0.01 (0.05) | 0.796 | 0.796 | 0.03 (0.02) | 0.062 | 0.309 |
| Agitation/aggression | 0.04 (0.03) | 0.101 | 0.919 | 0.10 (0.15) | 0.530 | 0.971 | -0.01 (0.03) | 0.850 | 0.991 | 0.00 (0.14) | 0.991 | 0.991 | -0.04 (0.03) | 0.167 | 0.919 |
| Depression/dysphoria | 0.06 (0.03) | 0.042 | 0.312 | -0.01 (0.12) | 0.931 | 0.931 | 0.01 (0.03) | 0.697 | 0.772 | -0.08 (0.12) | 0.505 | 0.753 | -0.06 (0.03) | 0.057 | 0.312 |
| Anxiety | 0.01 (0.03) | 0.606 | 0.634 | 0.11 (0.12) | 0.380 | 0.464 | 0.05 (0.03) | 0.157 | 0.367 | -0.24 (0.11) | 0.035 | 0.194 | 0.04 (0.03) | 0.266 | 0.423 |
| Elation/euphoria | -0.01 (0.01) | 0.658 | 0.875 | 0.09 (0.06) | 0.131 | 0.875 | -0.01 (0.01) | 0.707 | 0.875 | -0.06 (0.06) | 0.333 | 0.875 | 0.01 (0.01) | 0.622 | 0.875 |
| Apathy/indifference | 0.09 (0.03) | 0.001 | 0.015 | -0.14 (0.15) | 0.333 | 0.733 | -0.01 (0.03) | 0.806 | 0.887 | -0.07 (0.14) | 0.610 | 0.839 | -0.06 (0.03) | 0.039 | 0.216 |
| Disinhibition | 0.04 (0.02) | 0.040 | 0.270 | 0.03 (0.12) | 0.798 | 0.873 | 0.00 (0.03) | 0.874 | 0.873 | -0.02 (0.11) | 0.836 | 0.873 | -0.02 (0.02) | 0.354 | 0.873 |
| Irritability/lability | 0.04 (0.03) | 0.140 | 0.514 | 0.03 (0.15) | 0.860 | 0.860 | -0.04 (0.04) | 0.279 | 0.615 | -0.09 (0.15) | 0.534 | 0.839 | -0.04 (0.03) | 0.279 | 0.615 |
| Aberrant motor behavior | 0.03 (0.02) | 0.162 | 0.614 | 0.12 (0.14) | 0.405 | 0.782 | 0.05 (0.03) | 0.057 | 0.614 | -0.19 (0.14) | 0.168 | 0.614 | 0.00 (0.03) | 0.937 | 0.937 |
| Sleep | 0.09 (0.03) | 0.003 | 0.031 | 0.04 (0.13) | 0.790 | 0.946 | -0.02 (0.04) | 0.667 | 0.932 | -0.21 (0.12) | 0.088 | 0.324 | -0.02 (0.04) | 0.638 | 0.932 |
| Appetite | 0.06 (0.03) | 0.018 | 0.197 | -0.01 (0.11) | 0.954 | 0.954 | -0.02 (0.03 | 0.500 | 0.840 | -0.04 (0.10) | 0.665 | 0.840 | -0.03 (0.03) | 0.259 | 0.712 |

The data in the table represent the results of linear mixed effect models for raw NPI-Q total scores and subdomain scores at each time point, controlling for APOE4 carrier status, baseline dementia, baseline age, sex, and years of education as covariates. The primary predictors included the presence of LBD and LATE pathologies and their interaction terms with time. Results are presented with the estimate (β), standard error (SE), p-value, and q-value, corrected by the false discovery rate method. The presence of LB-NC and LATE-NC was not associated with longitudinal changes in any neuropsychiatric symptoms, whereas the total NPI-Q, apathy, and sleep scores increased with time.

Abbreviations: APOE4, apolipoprotein E ε4; LATE-NC, limbic-predominant age-related TDP-43 encephalopathy-related neuropathological changes; LB-NC, Lewy body-related neuropathological changes.
